# Microglia Ontology and Signaling

**DOI:** 10.3389/fcell.2016.00072

**Published:** 2016-06-29

**Authors:** Ayman ElAli, Serge Rivest

**Affiliations:** ^1^Neuroscience Laboratory, CHU de Québec Research Center (CHUL), Department of Psychiatry and Neuroscience, Faculty of Medicine, Laval UniversityQuebec, CA, Canada; ^2^Neuroscience Laboratory, CHU de Québec Research Center (CHUL), Department of Molecular Medicine, Faculty of Medicine, Laval UniversityQuebec, CA, Canada

**Keywords:** microglia origin, brain, developmental biology, signaling pathways, innate immunity

## Abstract

Microglia constitute the powerhouse of the innate immune system in the brain. It is now widely accepted that they are monocytic-derived cells that infiltrate the developing brain at the early embryonic stages, and acquire a resting phenotype characterized by the presence of dense branching processes, called ramifications. Microglia use these dynamic ramifications as sentinels to sense and detect any occurring alteration in brain homeostasis. Once a danger signal is detected, such as molecular factors associated to brain damage or infection, they get activated by acquiring a less ramified phenotype, and mount adequate responses that range from phagocyting cell debris to secreting inflammatory and trophic factors. Here, we review the origin of microglia and we summarize the main molecular signals involved in controlling their function under physiological conditions. In addition, their implication in the pathogenesis of multiple sclerosis and stress is discussed.

## The origin of microglia

The name microglia is derived from micro (i.e., small) and glia (i.e., glue). Resident microglia are mononuclear phagocytes and constitute the main resident immune cell population of the brain, representing up to 10% of the cells in the brain (Soulet and Rivest, 2008). Microglia were firstly visualized and identified in 1919 (reviewed in Tremblay et al., 2011, 2015), and despite the extensive research conducted since then, the origin of microglia is still elusive. Several different origins have been proposed for these cells. For instance, it has been proposed that microglia are derived from progenitors that originate from the neuroectoderm and/or the mesoderm, which colonize the brain from the early embryonic stage and throughout the fetal development stage (Chan et al., 2007; Soulet and Rivest, 2008). In addition, it has been proposed that microglia are derived from circulating blood monocytes, especially from the late gestational stage throughout the early post-natal stage (Chan et al., 2007). Despite these different proposed origins, a consensus view holds that microglia are derived from myeloid progenitors that infiltrate the brain during the different brain developmental stages. It is noteworthy to mention that ~95% of the microglia population are generated post-natally and more precisely after the formation of the blood–brain barrier (BBB). Once again, how these cells are maintained in the adult brain during lifespan is a matter of debate. One major view suggests that microglia are maintained in the adult brain through either a self-renewal process or by the expansion of a reservoir of progenitors that have colonized the developing brain (Soulet and Rivest, 2008). Another major view suggests that microglia are maintained in the adult brain through a turnover process by the differentiation of precursors present in the blood circulation, namely monocytes, that infiltrate the adult brain (Soulet and Rivest, 2008). However, the relevance of this turnover process under physiological condition is still unclear.

**Figure 1:**
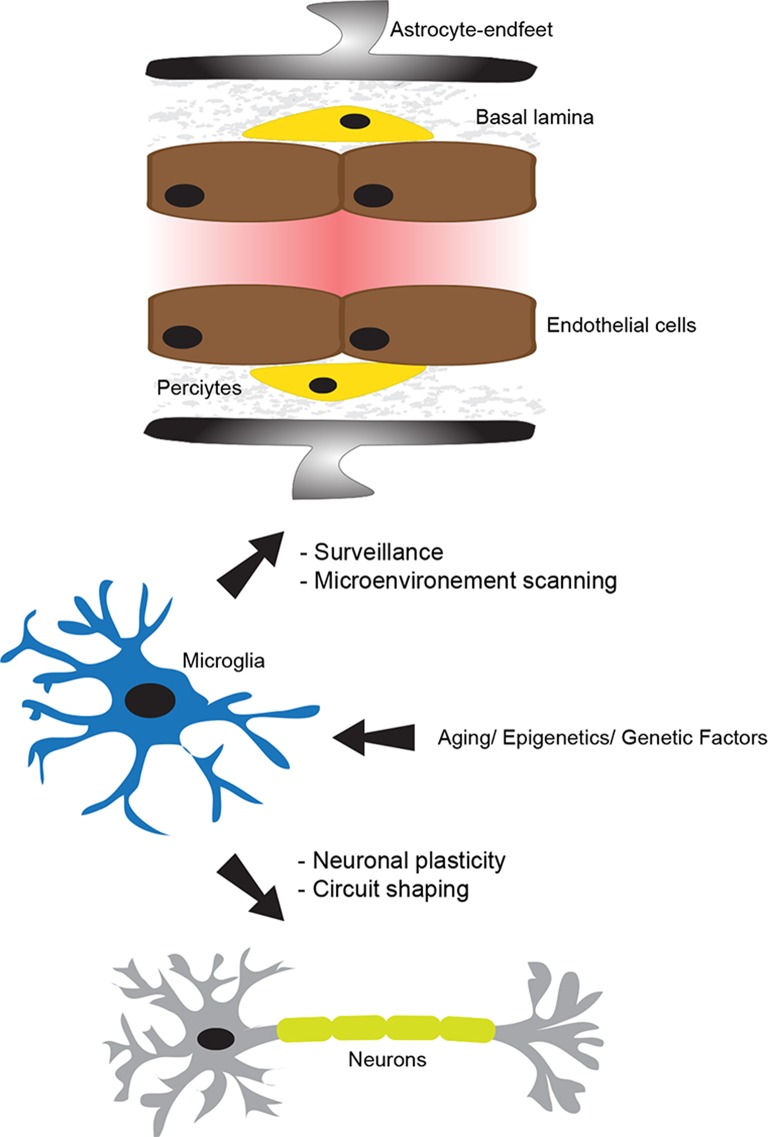
**The physiological role of microglia**. The neurovascular unit (NVU), which is constituted of tightly sealed endothelial cells, basal lamina, pericytes, astrocyte-endfeet, microglia, and neurons, plays a central role in maintaining brain homeostasis and functioning. In the non-neuropathological conditions, microglia use their dynamic processes to constantly survey and screen the microenvironment within the NVU. Microglia are fully equipped to detect a wide range of signals associated to an altered brain homeostasis. In addition, they actively and dynamically communicate with neurons and play an important role in supporting their functions. Importantly, their physiological role in the intact brain can be altered by several intrinsic factors, such as aging, epigenetics, and genetics. This figure has been adapted from ElAli and Rivest (2015).

Regardless their exact origin, it is widely accepted now that once microglial progenitors have infiltrated the brain, they adopt a highly ramified morphology (Kettenmann et al., 2011). Early reports suggested that this phenotype translates a resting state of these cells. This ramified phenotype is characterized by the presence of long cellular branching processes and a small cell body. While the cellular branches are constantly moving, the cell body remains in place. Recent findings revolutionized our understating for the function of microglia by showing that these cells are never resting and are continuously patrolling the brain in quest of any alteration in cerebral homeostasis (Kettenmann et al., 2013; Figure 1). More precisely, these recent findings showed that microglia use their dynamic and motile cellular processes as sentinels to survey and scan the microenvironment within their vicinity to detect any occurring change in brain homeostasis (Kettenmann et al., 2013). Microglia rapidly get activated in the presence of a threat and they adopt a less ramified and more amoeboid phenotype with a large soma. Following activation, these cells rapidly mount appropriate responses in order to deal with the emerging threat, which could range from phagocytosis to the release of molecular mediators, including immune and non-immune factors (Kettenmann et al., 2013; Lampron et al., 2013a). The new technical advances, especially intravital two-photon imaging in live animals, shed the light on the fascinating nature of microglia and outlined a more sophisticated role fulfilled by these cells in maintaining the brain tissue integrity under both physiological and pathophysiological conditions, by detecting both non-pathological- and pathological-derived molecular cues (Davalos et al., 2005; Nimmerjahn et al., 2005). These new studies showed for instance that microglia continuously scan the microenvironment within their vicinity by accurately and significantly modulating the length and the direction of their processes without detecting significant movement of their cell bodies. Under physiological conditions, these studies revealed how microglia through their processes make what seem to be specific and repeated cell–cell contacts, namely with neurons, astrocytes, and endothelial cells (Nimmerjahn et al., 2005; Figure 1). These functions are potentiated under pathophysiological conditions, where a focal and limited laser-induced cerebral damage stimulated the extension of microglial cell processes toward the injury site in few minutes (Davalos et al., 2005). In addition, even microglia that were not in a direct contact with the damaged area detected the damage, which led to their rapid mobilization toward the injury site (Davalos et al., 2005). Taken together, these reports highlight the dynamic nature of microglia and new insights into their functions.

## Signaling in microglia

**Figure 2:**
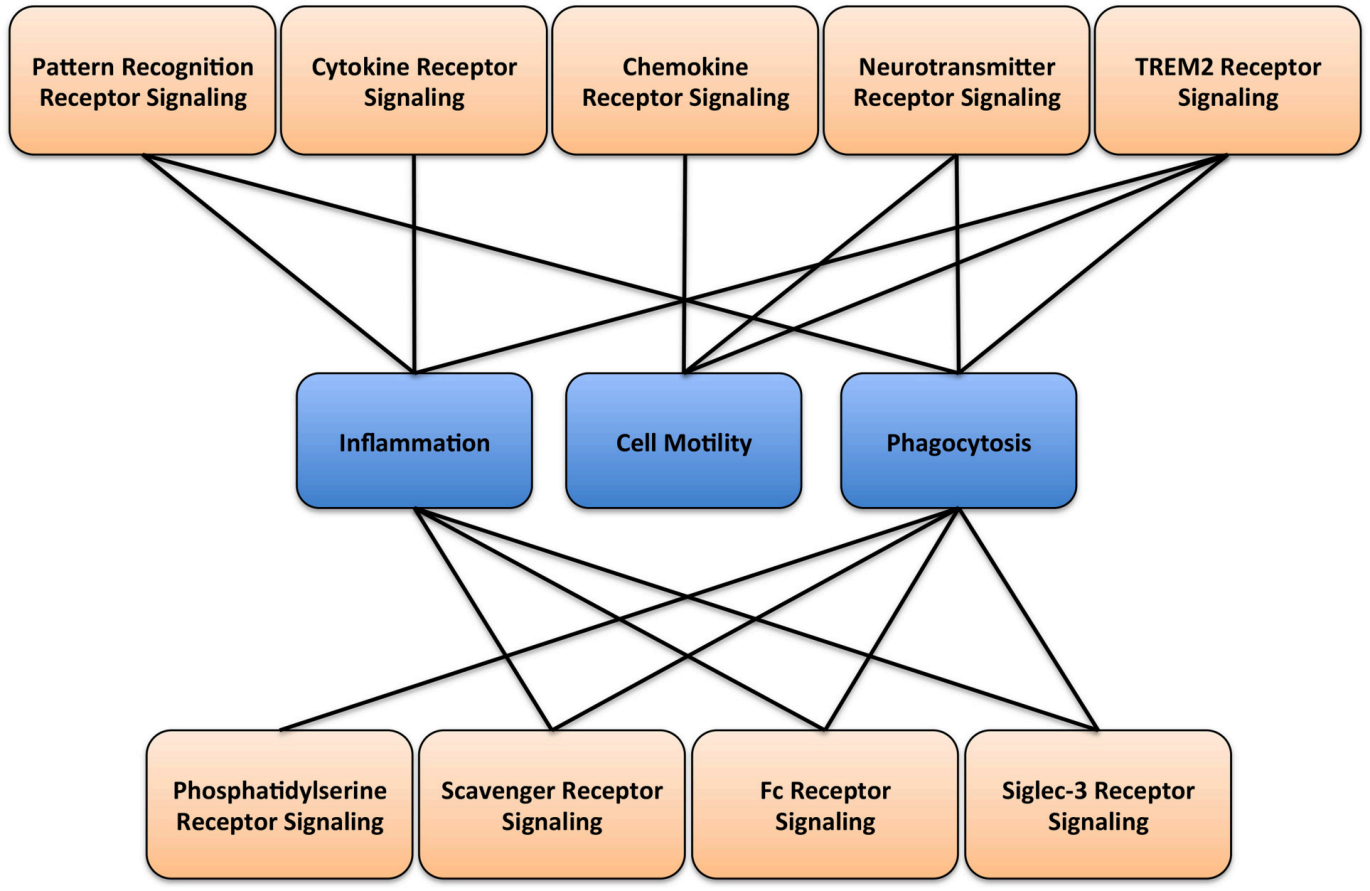
**Microglial signaling and function**. A diagram illustrating the main receptor-mediated signaling associated with respective microglial cellular functions.

Microglia are highly dynamic and undergo extreme remodeling process throughout lifespan. For example, it has been suggested that the concerted movements of microglial cell processes could survey the entire brain every couple of hours (Nimmerjahn et al., 2005). Importantly, this behavior requires a tight controlled regulation of the movement of cell processes, cell motility, cell morphology, phagocytosis, immune functions, and the secretion of molecular mediators. These functions are tightly and precisely controlled by several signaling pathways (Figure 2). Here, we will briefly present major signaling pathways that play essential roles in controlling key functions of microglia.

### Pattern recognition receptor signaling

Being the major line of defense in the brain, microglia express several receptors involved in the control of innate immune functions, especially the pattern recognition receptors (PRRs) that regroup three major receptor families, toll-like receptors (TLRs), nucleotide-binding oligomerization domain (nod)-like receptors (NLRs), and the retinoic acid-inducible gene-1 (RIG1)-like receptors (RLRs). These receptors recognize specific ligands, or “patterns,” called pathogen-associated molecular patterns (PAMPs) and danger-associated molecular patterns (DAMPs). These patterns include protein molecules, such as exogenous peptidoglycans and endogenous heat shock proteins, and non-protein molecules, such as adenosine triphosphate (ATP) and nucleic acid molecules (Kumar et al., 2011). Activation of these receptors induces specific signaling pathways that are involved in modulating microglial functions. TLR activation stimulates the recruitment of specific intracellular adaptor proteins, namely the myeloid differentiation primary response gene-88 (Myd88) that leads mainly to the induction of nuclear factor-kappa B (NF-κB) signaling pathway, and toll/interleukin-1 receptor (TIR)-domain-containing adapter-inducing interferon-β (TRIF) that leads mainly to the induction of interferon regulatory factor-3 (IRF3) signaling pathway. It is noteworthy to mention that among the 11 TLRs in humans and 13 in rodents, only TLR3 and TLR4 seem to signal via TRIF (Rivest, 2009; Lampron et al., 2013a). Until now, 21 NLRs have been identified, among which NLR pyrin domain containing (NLRP)1/2/3/6/10 and NOD1/2 are highly expressed in microglia (Rosenzweig et al., 2011). NLRP3 and NOD2 are the most studied receptors. NLRP3 activation mediates its oligomerization and stimulates the formation of a caspase-1 containing inflammasome that trigger the production of the active form of interleukine-1β (IL1β), which in turn activates downstream signaling pathways (Zambetti et al., 2012). On the other hand, NOD2 activation has been shown to stimulate the recruitment of the adaptor protein receptor-interacting serine/threonine-protein kinase-2 (RICK) that leads mainly to the induction of NF-κB signaling pathway (Ribes et al., 2012). Finally, RLR activation stimulates the recruitment of intracellular adaptor proteins, namely mitochondrial antiviral-signaling protein (MAVS) and tumor necrosis factor (TNF) receptor associated factor (TRAF), both of which trigger NF-κB signaling pathway (Yoneyama and Fujita, 2010). In addition, triggering RLRs could lead to the induction of IRF3 signaling pathway (Yoneyama and Fujita, 2010). In summary, PRR signal transduction converges mainly on two major signaling pathways, namely NF-κB and IRF3.

### Cytokine receptor signaling

Microglia express a wide range of cytokine receptors that play a crucial role in modulating microglial cell function. They also produce a large repertoire of cytokines that are essential in mediating cell functions. Among these cytokines, TNFα, transforming growth factor-β (TGFβ), and interleukin signaling pathways play major roles. Microglia express both TNF receptor subtypes, TNF receptor 1 (TNFR1/p55) and TNF receptor 2 (TNFR2/p75), and are, in parallel, a major source of TNFα in the brain (Dopp et al., 1997). It has been demonstrated that TNFR1 has a higher binding activity for soluble TNFα compared to TNFR2. On the other hand, TNFR2 has higher affinity to the membrane-anchored TNFα, which has been shown to play an important role in cell crosstalk (Veroni et al., 2010). Although both receptors have similar extracellular domains, their intracellular domains are structurally quite different, which translate the capacity of both receptors to trigger distinct and overlapping signaling pathways. TNFR1 and TNFR2 activation stimulates the recruitment of intracellular adaptor proteins that mediate the activation of downstream signaling pathways (MacEwan, 2002). Among these adaptor proteins, TNF receptor-associating factors (TRAFs) play an important role in orchestrating the formation of an intracellular signaling complex (MacEwan, 2002). Activation of both receptors triggers mainly the NF-κB signaling pathway (Sriram et al., 2006). Interestingly, several reports have demonstrated that TNFR1 exacerbates the inflammation (Longhi et al., 2013), whereas TNFR2 is neuroprotective (Marchetti et al., 2004). TNFR activation increases the release of TNFα by microglia, which creates a positive autocrine regulatory mechanism that actively participates in microglial cell activation (Nadeau and Rivest, 2000; Kuno et al., 2005).

In contrast to TNFα that plays an important role in potentiating microglial pro-inflammatory responses, TGFβ has been shown to play a role in counter-regulating these responses (Suzumura et al., 1993). TGFβ is a multifunctional cytokine that binds TGFβ receptor type II (TGFRII) and stimulates the recruitment of TGFβ receptor type I (TGFRI). Initially, this cytokine is released as inactive complex, from which the active form is released through different processes (Feng and Derynck, 2005). Interestingly, the CD36 receptor, which is abundantly expressed on microglia (El Khoury et al., 2003), has been shown to contribute in such an activation process (Yehualaeshet et al., 1999). Upon activation, the TGFRI/RII complex receptor triggers the formation of Small and Mothers Against Decaplentaplegic-2/3/4 (SMAD-2/3/4) protein complex that translocates into the nucleus to regulate the expression of several target genes (Feng and Derynck, 2005). In parallel, TGFβ mediates its effects through another signaling pathway, independently of SMADs, which results in the activation of mitogen-activated protein kinase (MAPK) and Rho GTPases signaling pathways (Engel et al., 1999). TGFβ signaling pathway has also been shown to reduce the production of IL6, IFNγ, and several chemokines, namely monocyte chemoattractant protein-1 [MCP1; i.e., chemokine ligand 2 (CCL2); Letterio and Roberts, 1998] as well as the production of nitric oxide (NO) and the formation of reactive oxygen species (ROS; Vodovotz et al., 1993).

Microglia express several IL receptors (ILRs), namely IL1Rs [e.g., IL1 type I receptor (IL1RI), IL1 type II receptor (IL1RII), and IL1 receptor accessory protein (IL1RAcP)], IL5R, IL6R, IL8R, IL9R, IL10R, IL12R, IL13R, and IL15R. IL1β and IL6 play particularly important roles in the microglial cell function. IL1β signaling pathway potentiates microglial pro-inflammatory responses. This cytokine is essentially produced in the early stages of the innate immune response engagement, in response to TLR/Myd88/NF-κB activation (Herx et al., 2000). IL1β is initially produced as an inactive protein, from which the active form is generated via a proteolytic process that includes the formation of an inflammasome (John et al., 2005). IL1β activates a receptor complex formed by IL1RI and IL1RAP (Steinman, 2013), thus inducing NF-κB signaling pathway (Spörri et al., 2001). IL6 is considered as a pro-inflammatory cytokine that upon binding IL6R stimulates the recruitment of gp130 receptor (Erta et al., 2012). The receptor complex activates several intracellular signaling pathways, namely signal transducer and activator of transcription (STAT) pathway, MAPK pathway, and phosphatidyl inositol-3 kinase (PI3K) pathway (Erta et al., 2012). The role of IL6 in the brain is complex and not yet completely understood. Finally, IL4 and IL10 are potent anti-inflammatory molecules that have been shown to deeply affect microglial cell function (Ledeboer et al., 2000).

### Chemokine receptor signaling

Chemokines are small chemotaxic cytokines that possess the ability to induce chemotaxis in nearby responsive cells. Chemokines are a large family of molecules that are characterized by the presence of conserved cysteine residues in their N-terminal sequences. Based on the spacing of their first two cysteine residues, they are classified into four distinct subgroups, C chemokines (i.e., one N-terminal cysteine), CC chemokines (i.e., two adjacent N-terminal cysteines), CXC chemokines (i.e., one amino acid between the two N-terminal cysteines), and finally CX3C chemokines (i.e., three amino acids between the two N-terminal cysteines; Fernandez and Lolis, 2002). Chemokines are produced in the brain by different populations of cells, such as neurons and microglia. All chemokines mediate their effects following their release as soluble molecules that generate a chemotactic gradient for cell mobilization and migration, except CX3C ligand 1 (CX3CL1; i.e., fractalkine) that mediates its effect either as a soluble molecule or a membrane-anchored molecule (Fernandez and Lolis, 2002). Depending on the nature of the stimuli, the chemokines are functionally divided into two groups, homeostatic, which are constitutively produced and contribute in basal cell migration, and inflammatory, which are induced once the inflammatory response is engaged (Sokol and Luster, 2015). Microglia express a wide range of chemokine receptors with different expression levels that are specie- and region-dependent, namely CCL1 receptor (CCR1), CCR2, CCR3, CCR4, CCR5, CCR6, CCR7, CXCR1, CXCR2, CXCR3, CXCR4, and CXCR5 (reviewed in Kettenmann et al., 2011). Chemokine receptors are G protein-coupled receptors (GPCRs), which upon activation modulate different downstream signaling pathways that lead to the regulation of calcium (Ca^2+^) homeostasis, Rho GTPases activity, and MAPK signaling pathway. The CCL2/CCR2, CCL5 [i.e., regulated on activation, normal T cell expressed and secreted (RANTES)]/CCR5, and CX3CL1/CX3CR1 signaling pathways play particularly important roles in microglial cell function. Following TLR activation, CCL2 is produced by microglia, thus acting in an autocrine and paracrine manner. In parallel, the CCL2/CCR2 signaling pathway plays a central role in regulating peripheral leukocyte recruitment and infiltration into the brain during inflammatory reactions (El Khoury et al., 2007). Similar to CCL2/CCR2 signaling pathway, CCL5/CCR5 signaling pathway is regulated in an autocrine and paracrine manner. Activation of this signaling pathway has been reported to suppress the production of major pro-inflammatory cytokines, namely TNFα, IL1β, and IL6 (Gamo et al., 2008). On the other hand, CX3CL1 is secreted essentially by neurons and binds to its receptor CX3CR1 in microglia, thus acting as messenger that is involved neuron-microglia communication. The CX3CL1/CX3CR1 signaling pathway plays an important role in regulating microglial dynamics and migration, which is central for surveying neuronal function through lifespan (Paolicelli et al., 2014). In parallel, the CX3CL1/CX3CR1 signaling pathway has been shown to control the microglial production of several pro-inflammatory cytokines, namely TNFα, IL1β, and IL6 (Limatola and Ransohoff, 2014). Interestingly, inactivation of both CCL5/CCR5 and CX3CL1/CX3CR1 signaling pathways was found to impair microglial migration and communication between neurons and microglia. This may result in excessive inflammatory response and neuronal damage (Gamo et al., 2008; Cho et al., 2011).

### Neurotransmitter receptor signaling

Microglia express a wide range of different types of neurotransmitter receptors that play central role in different cell functions, namely by mediating the interaction and crosstalk between microglia and neurons in physiological and pathological conditions. Among these neurotransmitter receptors, purinoceptors, glutamate receptors, cholinergic receptors, adrenergic receptors, and dopamine receptors have been shown to play essential roles in modulating microglial cell activity (reviewed in Kettenmann et al., 2011). The purinoceptor signaling pathways play a central role in microglial cell function due to the biological significance of their natural ligands, the nucleotides. The extracellular neuronal release of nucleotides, such as ATP, ADP, uridine triphosphate (UTP), and UDP, translates a major cell dysfunction that precedes or accompanies cell death, which is perceived by microglia as alerting signals (Illes and Alexandre Ribeiro, 2004; Burnstock and Verkhratsky, 2010). These extracellular nucleotides are recognized by several purinoceptors, namely the metabotropic P1 adenosine receptors GPCRs, ionotropic P2X purinoceptors (ligand-gated cationic channels), and metabotropic P2Y purinoceptors (GPCRs; reviewed in Kettenmann et al., 2011). These receptors have the ability to modulate microglial mobility and shape (Xiang et al., 2006; Färber et al., 2008), which may result in deep changes in membrane current and Ca^2+^ homeostasis (Boucsein et al., 2003; Light et al., 2006). Ca^2+^ homeostasis regulates several downstream signaling pathways in microglia, including those involved in cell motility and phagocytosis (Hoffmann et al., 2003). In addition, purinoceptors are able to modulate innate immune functions in the CNS (reviewed in Kettenmann et al., 2011).

Among the glutamate receptors expressed in microglia, the ionotropic α-Amino-3-hydroxy-5-methyl-4-isoxazolepropionic acid (AMPA) receptor and the metabotropic glutamate receptors (mGluRs) are the best characterized. These receptors are activated by glutamate released from neurons and regulate Ca^2+^ homeostasis, NF-κB signaling pathway, and TNFα turnover (Noda et al., 2000; Kaushal and Schlichter, 2008). The gamma-aminobutyric acid (GABA) constitutes the major inhibitory neurotransmitter in the brain (Harris and Allan, 1985). The metabotropic G protein-coupled receptor GABA_B_ type is expressed in a subpopulation of microglia and modulates Ca^2+^ homeostasis and down regulates the release of IL6 (Kuhn et al., 2004). α7 nicotinic (α7nAChRs) are the best characterized acetylcholine receptors in microglia, which seem to have anti-inflammatory effects (De Simone et al., 2005). Adrenergic receptors regulate Ca^2+^ homeostasis and reduce the release of TNFα, IL6, and NO by microglia (Färber et al., 2005). Functional D1/2-like dopamine receptors have been identified in microglia and they seem to modulate potassium (K^+^) homeostasis and cytokine production (Färber et al., 2005). Recent experimental findings have provided evidence that neurotransmitter signaling pathways may result in microglial cell mobility (Fontainhas et al., 2011).

### TREM2 receptor signaling

Insights into the functions of triggering receptor expressed on myeloid cells-2 (TREM2) receptor are recently emerging. TREM2 receptor is mainly expressed in dendritic cells (DCs), osteoclasts, and microglia (Colonna, 2003). It is a cell surface receptor that belongs to the immunoglobulin (Ig) superfamily family that is encoded by a gene cluster linked to major histocompatibility complex (MHC). TREM2 is a transmembrane receptor that lacks cytoplasmic signaling element, which is replaced by a charged domain that stimulates its association with other transmembrane adaptor proteins, namely DNAX-activation protein-12 (DAP12; Colonna, 2003). The association of both receptor triggers microglia immunoreceptor tyrosine-based activation motif (ITAM) signaling pathway that leads to cytoskeleton reorganization, chemokine production, and phagocytosis stimulation, which is accompanied by a reduction in pro-inflammatory cytokine and ROS generation (Fu et al., 2014). Interestingly, the depletion of TREM2 in mice has been shown to severely impair microglial cell response to microenvironment stimuli, morphology, and migration, thus outlining an important role of this receptor in microglial cells (Rivest, 2015; Wang et al., 2015). Activation of TREM2 signaling pathway is induced upon the biding of several charged lipid derivatives, including phospholipids (Wang et al., 2015). More precisely, TREM2 seems to act as a sensor for several anionic and Zwitter-ionic lipids that are derived from the cell membrane of neurons and glial cells, as lipid derivatives generated from some organelles such as mitochondria failed to trigger TREM2 activation (Wang et al., 2015). TREM2 is able to bind the heat shock protein-60 (Hsp60), which enhances phagocytosis by these cells (Stefano et al., 2009).

A recent genome-wide association studies (GWAS) have shown that a rare Arginine-47-Histidine (R47H) mutation of TREM2 is associated with a substantial increase in the risk of developing Alzheimer's disease (AD; Jonsson et al., 2013). The significance of this mutation has been recently confirmed experimentally when TREM2 depletion in 5XFAD mice significantly increased cerebral amyloid beta (Aβ) load due to a dysfunctional response of microglia, which fail to cluster around Aβ plaques and become apoptotic (Wang et al., 2015). Importantly, in this study the authors demonstrated that TREM2 acted as a sensor of several modified lipids known to associate with fibrillar Aβ in lipid membranes and to be exposed on the surface of damaged neurons, which is in line with the implications of the R47H mutation that impairs essentially TREM2 detection of lipid ligands (Wang et al., 2015). However, the implication of the cerebral Aβ reduction on cognition has not been addressed, thus the functional outcome of the here above reported observations cannot be assessed (Rivest, 2015).

### Phosphatidylserine receptor signaling

Phosphatidylserine (PS) is a phospholipid that is normally sequestered in the inner leaflet of plasma membrane, which is exposed once cells are undergoing apoptosis (Martin et al., 1995). The recognition of PS by specialized receptors (PSRs) is crucial for an efficacious clearance of cell debris by specialized phagocytes (Ravichandran, 2011). Microglia express several specialized PSRs that directly bind PS, namely brain-specific angiogenesis inhibitor-1 (BAI1), T-cell immunoglobulin mucin receptor 1 (TIM1), and TIM4 (Ravichandran, 2011). In addition, PSRs include some receptors that indirectly bind PS by using an intermediate molecule, namely c-mer proto-oncogene tyrosine kinase (MerTK) that uses growth arrest specific gene-6 (Gas6) as a bridging molecule, and vitronectin receptors (i.e., αvβ3 or αvβ5 integrins) that use milk fat globule-EGF factor 8 protein (MFG-E8) as a bridging molecule (Ravichandran, 2011). The direct and indirect recognition of PS by PSRs are crucial in mediating apoptotic cell phagocytosis by microglia. Importantly, the cooperation and activity coordination among these receptors seem to be central in mediating an efficacious phagocytosis (Mazaheri et al., 2014). For example, BAI1 has been reported to be in charge of controlling the formation of phagosomes around dying neurons and the transport of apoptotic cargos, while TIM4 is more involved in the downstream stabilization of phagosomes (Mazaheri et al., 2014). However, both receptors are required to mediate a successful engulfment of apoptotic neurons (Mazaheri et al., 2014). Besides being central in microglial cell phagocytosis, PSR activation is accompanied by a significant reduction in the release of several pro-inflammatory molecules, such as TNFα, IL1β, and NO (De et al., 2002).

### Scavenger receptor signaling

Scavenger receptors (SRs) comprise structurally diverse cell membrane receptors that have been shown to contribute to several cell functions, namely adhesion, and uptake of negatively charged macromolecules, and oxidized or acetylated low-density lipoprotein (LDL; Canton et al., 2013). Interestingly, several classes of SRs are expressed in microglia and they play key roles in innate immunity (Peiser et al., 2002). Among these SRs, macrophage SR class AI (SR-AI), macrophage receptor with collagenous structure (MARCO), SR-B3 (i.e., CD36), macrosialin (i.e., CD68), and recently lectin-like oxidized low-density lipoprotein receptor-1 (LOX1) have been reported to play particularly important roles in microglial cell function. SR-AI is a trimer integral glycoprotein composed of a cytosolic tail, a transmembrane region, a spacer domain, α-helical coiled-coil motif, collagen-like domain, and a cysteine-rich C-terminal domain (Matsumoto et al., 1990). SR-AI seems to bind a wide range of molecules, namely modified lipids and proteins, polyribonucleotides, polysaccharides, and anionic phospholipids (de Winther et al., 2000). SR-AI activation induces production of a several inflammatory factors, namely TNFα, IL1β, IL-6, and NO (Coller and Paulnock, 2001). Although MARCO is a member of the SR-A family, this receptor binds additional distinct ligands, outlining a larger role in regulating cell function (Thakur et al., 2009). Indeed, MARCO activation triggers microglial cytoskeleton reorganization, a mechanism that is highly important in the process of phagocytosis and cell mobility (Granucci et al., 2003). CD36 is a member of the SR-B family that is abundantly expressed in microglia (Coraci et al., 2002). It binds a wide range of ligands, such as native/oxidized LDL, collagen, thrombospondin, oxidized phospholipids, and long-chain fatty acids (Febbraio et al., 2001). Stimulation of CD36 signaling pathway enhances microglial cell phagocytosis and production of pro-inflammatory cytokines, chemokines, and ROS (El Khoury et al., 2003). CD36 has also been shown to promote the formation of a TLR4/6 heterodimer and engagement of both MyD88 and TRIF adaptors, which ultimately affect microglia functions (Stewart et al., 2010). CD68 is a member of the SR-B family that is characterized by the presence of a mucin-like motif in the extracellular domain (Kobayashi et al., 2013). It is mainly expressed on the membrane of intracellular late endosomes under normal conditions, and its expression increases following activation by oxidized LDL (Ramprasad et al., 1996). CD68 is actively involved in microglial cell phagocytosis (Perego et al., 2011). LOX1 is found as a homodimer on cell membrane, and is the only identified member of SR-E family, which has been shown to primarily bind and process oxidized LDL (Reiss et al., 2009). Recent findings have revealed that the LOX1 structure comprises a reactive backbone that can bind to a wide range of ligands, including small molecules, such as acidic phospholipids, and whole cell structure, such as apoptotic cells and bacteria (Honjo et al., 2003). Although LOX1 was originally identified as endothelial receptor for oxidized LDL (Sawamura et al., 1997), recent reports showed that this receptor is also expressed on microglial cells (Zhang et al., 2012). LOX1 activation induces NF-κB signaling pathway that leads to the release of several pro-inflammatory cytokines and ROS (Zhang et al., 2012; Li et al., 2013). Importantly LOX1 is necessary for microglia for sensing diffuse signals generated by neuronal injury, such as Hsp60 (Zhang et al., 2012). Hsp60 acts as an endogenous LOX1 ligand (Delneste et al., 2002). These data suggest that LOX1 shares some functional features with TLRs, outlining the implication of this receptor in regulating brain immune responses. However, more studies are warranted to better address this point.

### Fc receptor signaling

Fc receptors (FcRs) belong to the immunoglobulin (Ig) superfamily that binds the constant domain (Fc) of Ig. They are subdivided into different subclasses based on their binding to specific isotype classes and subclasses of Ig; FcαR binds IgA, FcδR binds IgD, FcμR binds IgM, FcεR binds IgE, and FcγR binds IgG (Daëron, 1997). Interestingly, microglia express all FcR subgroups (Okun et al., 2010). The cytoplasmic tail of FcRs could contain ITAM that provides a positive signal for cell activation, and immunoreceptor tyrosine-based inhibition motif (ITIM) that provides a negative signal for cell activation. As such, the outcome of the receptor aggregation is totally dependent on the immunoreceptor tyrosine-based motif that was engaged (Daëron, 1997). Typically, FcR activation results in their aggregation on cell surface, and consequently triggering ITAM signaling pathway that leads to the sequential activation of Src and Syk tyrosine kinases (Daëron, 1997). This results in triggering several downstream signaling pathways, such as NF-κB and MAPKs (Song et al., 2004). This pathway triggers deep changes in microglial cell function and behavior, mainly by modulating the release of cytokines and by enhancing their phagocytic capacity (Okun et al., 2010). In some cases, FcR aggregation on cell surface triggers ITIM signaling pathway involving Src homology region 2 domain-containing phosphatase-1 (SHP1) and Src homology region 2 domain-containing inositol phosphatase-1 (SHIP1), which is a potent inhibitory mechanism for microglia (Billadeau and Leibson, 2002). In parallel, FcRs have been shown to signal independently of their binding to Ig. For instance, it has been reported that soluble FcRs can modulate the release of some cytokines, such as TNFα and IL6 (Okun et al., 2010). However, more studies are warranted to better address the implication of this non-Ig dependent signaling pathway in regulating microglial cell function under both physiological and pathophysiological conditions.

### Siglec-3 receptor signaling

The sialic acid-binding immunoglobulin-type lectin-3 (Siglec-3; i.e., CD33) is a type 1 transmembrane receptor that is essentially expressed on the surface of cells that belong to the myeloid lineage (reviewed in Crocker et al., 2007). CD33 is a member of the CD33-related Siglecs, which has two extracellular immunoglobulin domains (IgV/IgC2) that recognize sialic acid residues of glycoproteins and glycolipids, and an intracellular domain that contains ITIM in humans, or ITIM-like domain in rodents (Crocker et al., 2007). ITIM and ITIM-like domains act as docking sites or SHP1 and SHP2, via which CD33 and CD33-related Siglecs execute several inhibitory functions on cell adhesion, endocytosis, and cytokine release by immune cells (Crocker et al., 2007). CD33 is expressed by microglia in both the human and rodent brains, which upon activation significantly reduces microglial cell phagocytic capacity (Griciuc et al., 2013). In addition, Siglec-E, a CD33-related Siglecs has been recently shown to recognize neural glycocalyx, and to inhibit the phagocytosis of neural debris by microglia *in vitro* (Claude et al., 2013). Taken together, these reports clearly outline the central role of CD33 and CD33-related Siglecs in acting as regulatory receptors that tightly control the immune functions of microglia under both physiological and pathophysiological conditions. However, more studies are warranted in order to fully address their role in the brain.

### Miscellaneous receptor signaling

Microglia express several other set of receptors that contribute, to different extents, in controlling and regulating their functions. The complement receptors (CRs) play a central role in the innate immune response (Crehan et al., 2012, 2013). Macrophage colony-stimulating factor receptor (m-CSFR) stimulates the phagocytic capacity of microglia, which is a critical mechanism for the clearance of amyloid beta from the CNS (Boissonneault et al., 2009). Sigma-1 receptor (S1R) is a membrane-associated protein that binds endogenous monoamine molecules, which affect Ca^2+^ homeostasis and suppress several key features of microglia (Hall et al., 2009). In addition, the progesterone receptor membrane component-1 (PGRMC1) has been recently identified as the putative S2R binding site (Xu et al., 2011). Interestingly, the S2R/PGRMC1 has been shown to be activity involved in regulating microglial cell activity, which had implications on neurite outgrowth (Bali et al., 2013). Finally, the OX2 (CD200) cell membrane glycoprotein receptor (CD200R), a member of the Ig superfamily, plays a role in controlling microglial cell activity (Hoek et al., 2000).

The receptor for advanced glycation endproducts (RAGE) is a multiligand receptor that belongs to the Ig superfamily, which consists of an extracellular domain, a single transmembrane domain and a cytosolic domain (Schmidt et al., 2001). It binds to a wide range of ligands, such as advanced glycosylation endproducts (AGE), S100/calgranulin family of proteins, and high mobility group box-1 (HMGB1; Schmidt et al., 2001). RAGE is expressed in microglia and induces NF-κB and MAPK signaling pathway, which causes the release of several pro-inflammatory cytokines, such as TNFα and IL1β (Fang et al., 2010). Interestingly, RAGE mediates some of these effects by interacting with the chemotaxic GPCRs, formyl peptide receptor-1/2 (FPR1/2; Slowik et al., 2012). LDL related receptor-1 (LRP1) is a cell membrane multiligand receptor composed of an extracellular domain, a transmembrane domain and an intracellular domain that contains two NPXY motif copies involved in signal transduction (Herz and Strickland, 2001). LRP1 is an intriguing receptor due to its diverse roles in multiple unrelated biological and cellular processes, such as endocytosis, cell–cell communication, cytoskeleton reorganization, intracellular signaling, lipid homeostasis, and clearance of apoptotic cells (Lillis et al., 2008). LRP1 has an extremely wide range of various ligands that include lipoproteins, enzymes, protein complexes, endotoxins, viral proteins, and apoptotic cells (Herz and Strickland, 2001; Gardai et al., 2005). In parallel, LRP1 has been reported to interact with several other membrane receptors, namely APP, platelet-derived growth factor receptor-β (PDGFRβ), and β2 integrins, the latter been implicated in LRP1-dependent cell migration (Ranganathan et al., 2011). Microglia express a high level of LRP1, which modulates several cell functions, such as phagocytosis and migration, via the regulation of janus kinase (JAK)/signal transducer and activator of transcription-1 (STAT1) and c-jun N-terminal kinase-1/2 (JNK1/2) signaling pathways, respectively (Pocivavsek et al., 2009; Jeon et al., 2012). Moreover, LRP1 signaling pathway provides potent anti-inflammatory processes in microglia (Overton et al., 2007; Gaultier et al., 2008). Taken together, these data outline an important role of LRP1 in modulating the inflammatory response by innate immune cells (Gorovoy et al., 2010; May et al., 2013).

## Role of microglia in brain disorders

Microglia have been shown to play important roles in brain disorders. Here, we will discuss recent reports that outlined a previously unrecognized role of these cells in different pathological conditions.

### Microglia in multiple sclerosis

Activation of microglia by lipopolysaccharide (LP) *in vivo* prevents the accumulation of myelin debris and avoids premature recruitment of oligodendrocyte (OD) progenitor cells (OPCs) that are restricted to the border of demyelinated regions (Glezer et al., 2006). The TLR4 ligand can induce these effects by modulating expression of key genes involved in OPC recruitment and differentiation, and in OD-mediated remyelination. In the course of an ongoing innate immune response, cells that express an oligodendrocyte transcription factor 1 (OLIG1), OLIG2, and proteolipid protein (PLP) are normally recruited to the demyelinated sites, whereas the presence of LPS increases the expression of *Pdgfr*α (platelet-derived growth factor receptor, α polypeptide). OLIG1, OLIG2, and *Pdgfr*α are key genes involved in OPC mobilization and remyelination process. Accordingly, LPS-induced microglia activation renders the brain tissue more responsive to myelin repair. This is also the case in response to the TLR2 ligand zymosan, which substantially increases myelination by transplanted OPCs in the retina (Setzu et al., 2006).

These data go against the proposed unfavorable role of brain inflammation in demyelinating diseases, such as multiple sclerosis (MS). However, although a single LPS injection is probably not enough to cause neurodegeneration and demyelination, long-term exposure to PAMPs, as it occurs during severe meningitis, does have profound effects on the nervous tissues. Indeed, OD death occurs in mixed cultures of glial cells that are chronically exposed to LPS, and hypomyelination occurs in developing white matter of immature rodents following intracerebral LPS injection (Lehnardt et al., 2002). Administration of LPS can also result in severe axonal and neuronal loss following sub-threshold hypoxic–ischemic insult that normally induces no discernable neuronal injury (Lehnardt et al., 2003).

Microglia has been shown to actively contribute to brain functioning by controlling the survival of developing neurons, the maintenance of developing and mature synapses, the maturation of neuronal circuits, and the shaping of neuronal connectivity (Tremblay et al., 2011). Importantly, microglia have been shown to densely populate the neurogenic zones, such as the subventricular zone (SVZ; Mosher et al., 2012), where they seem to be more activated compared to non-neurogenic zones (Goings et al., 2006). Interestingly, the presence of microglia in the SVZ has been demonstrated to play an important role in promoting neurogenesis and oligodendrogenesis via the production of cytokines (Shigemoto-Mogami et al., 2014). Indeed, new reports are suggesting that these innate immune cells play a central role in myelin turnover under physiological and pathophysiological conditions by phagocyting myelin debris and by influencing oligodendrogenesis. For instance, when demyelination occurs in the adult brain, microglia adopt a phenotype associated with phagocytosis and the recruitment of OPCs (Jurevics et al., 2002; Olah et al., 2012). The CX3CL1/CX3CR1 signaling pathway plays a key role in this physiological interaction between microglia and neurons (Paolicelli et al., 2011). CX3CL1 is secreted by neurons and binds to its receptor, CX3CR1, which is exclusively expressed on microglial cells in the healthy brain (Tremblay et al., 2011). The CX3CL1/CX3CR1 axis plays a crucial role in regulating microglial behavior and function, which decisively affect brain functioning (Thériault et al., 2015).

In this regard, our group has recently demonstrated that CX3CR1 depletion severely impeded myelin removal by microglia, thus exacerbating myelin debris deposition and spreading throughout the white matter of a mouse model of progressive MS induced by cuprizone-based diet (Lampron et al., 2015). This resulted in inefficient axonal remyelination characterized by aberrant myelin patterns, which demonstrates the critical role of CX3CR1 in promoting the clearance of degenerated myelin by microglial cells (Lampron et al., 2015). The knockout of CX3CR1 blocked the clearance of myelin debris by microglia, which greatly affected the integrity of axons and myelin sheaths, preventing proper remyelination. These results highlight the crucial role played by CX3CR1 in myelin removal and show that there can be no efficient remyelination following a primary demyelinating insult if myelin clearance by microglia is impaired. We also demonstrated a marked CCR2-dependent infiltration of bone marrow-derived cells (BMDCs) in demyelinating areas, but these cells do not impact demyelination and remyelination.

Myelin removal is a critical step in the remyelination process (Kotter et al., 2006). Cells of the mononuclear phagocytic system, including monocyte-derived macrophages (MDM) and microglia, are actively implicated in the clearance of myelin debris. While monocytes do not migrate into the CNS under normal conditions, we and others have shown the specific infiltration of MDMs under pathological conditions (Lampron et al., 2012, 2013b). Microglia and macrophages are regarded as detrimental in MS and experimental autoimmune encephalomyelitis (EAE), through their roles in autoimmunity such as antigen presentation and pro-inflammatory cytokine production. However, these noxious roles might mask other beneficial properties. During demyelination, microglia exert a phenotype associated with phagocytosis and the recruitment of OPC. While recent work unraveled differences between the roles played by blood-borne macrophages and microglia during autoimmune-mediated demyelination (Yamasaki et al., 2014), their respective functions in the process of primary de- and remyelination of the brain were not completely understood until recently.

Immunomodulators have been very efficient in the control of acute demyelinating events in relapsing-remitting courses of MS. However, these molecules have largely been ineffective in alleviating chronic demyelination in progressive forms of the disease, suggesting a primary neurodegenerative course (Stys et al., 2012). As autoimmunity does not seem to play a significant role in progressive forms of MS, a balance between limiting demyelination and boosting remyelination of affected sites must be reached for long-term therapeutic support of these patients (Fox et al., 2012). However, the results reported in our recent study propose important new concepts to take into consideration: there can be no efficient remyelination if microglia are unable to clear degenerate myelin from affected axons. As such, an optimal treatment strategy could consist in boosting microglial phagocytic processes while limiting inflammatory responses, combined with agents targeting OD physiology.

Activated microglia may stimulate or block oligodendrogenesis dependently on the nature of released cytokines in an animal model of MS (Butovsky et al., 2006). More precisely, high levels of IFNγ triggered microglial cells to adopt a phenotype that impeded oligodendrogenesis, which was associated with TNFα production. Whereas, IL-4 reversed these effects, and the injection of IL-4-activated microglial cells into the cerebrospinal fluid of mice resulted in increased oligodendrogenesis in the spinal cord and improved clinical outcomes (Butovsky et al., 2006). These results outlined a previously unknown role of microglia in influencing endogenous oligodendrogenesis via a selective production of cytokines (Butovsky et al., 2006). It has been shown that microglia exhibit a pro-inflammatory-like phenotype (i.e., M1 phenotype) during the recruitment of OPCs, which was followed by a switch toward an anti-inflammatory-like phenotype (i.e., M2 phenotype) during the maturation of OPCs (Boven et al., 2006; Miron et al., 2013). In addition, impaired myelin removal during demyelination and myelin debris accumulation alter CNS remyelination by affecting the differentiation of OPCs (Kotter et al., 2005, 2006).

### Microglia in stress

The exposure to stress induces a profound shaping of the neuronal network and deeply affects cognitive functions (Blandino et al., 2006). Interestingly, recent reports are suggesting that stress is causatively implicated in modulating the function of microglial cells, which play important roles in shaping the neuronal network in the adult brain. One of the initial reports has shown that stress significantly increases the expression of IL1β by microglial cells (Blandino et al., 2006). In addition, stress has been shown to stimulate the capacity of microglia to produce CCL2 and to increase the expression of TLR2 in these cells (Shimoda et al., 2006). Interestingly, stress was reported to influence microglial cell morphology as well. For instance, stress induced a specific form of microglial remodeling, which is translated by the formation of several new branching indicating a higher ramification rate (Hinwood et al., 2013).

Although the overwhelming experimental findings are demonstrating that stress directly influences microglial cell morphology and function (Walker et al., 2013), the underlying mechanisms are still emerging. There is a wide range of evidence demonstrating the ability of stress to significantly increase the levels of circulating corticosterone (Ulrich-Lai and Herman, 2009). Steroid hormones, namely glucocorticoids, have been shown to act as regulators of microglial inflammatory activity (Sierra et al., 2008). Recently, the CX3CL1/CX3CR1 signaling pathway has been shown to be implicated in regulating the interaction between neurons and microglial in mice exposed to stress conditions (Milior et al., 2016). Disturbing neuron-microglia communication by deregulating CX3CL1/CX3CR1 signaling pathway prevented the effects of chronic unpredictable stress on microglial function, neuronal plasticity, and depressive-like behavior (Milior et al., 2016).

Interestingly, high level of stress exposure has been linked to the severity of autism spectrum disorder (ASD) in children (Pardo et al., 2005). In parallel, several line of evidence demonstrated the presence of a sustained chronic neuroinflammatory response associated with microglial cell activation in the brain of children with ASD (Pardo et al., 2005). Indeed, the expression levels of several pro-inflammatory cytokines, such as IL6, IFNγ, and TNFα, were significantly increased in the brains of ASD patients compared with the controls (Vargas et al., 2005; Chez et al., 2007). It is supposed that the sustained chronic activation of microglia triggers the generation of a pro-inflammatory microenvironment, which contributes to brain underconnectivity due to loss of synaptic connections and neuronal death (Rodriguez and Kern, 2011). Based on these findings, it is plausible to speculate that by reducing brain inflammation it may be possible to improve the efficacy of early behavioral and learning interventions, and consequently enhance developmental outcomes of children with ASD. As such, future studies, which aim at investigating the effects of some pharmacological approaches that reduce microglial cell activation to mitigate ASD symptoms, are warranted.

## Concluding remarks

As mentioned above, microglia play an important role in maintaining brain's integrity and plasticity in normal conditions. Under these conditions, they are highly ramified cells, which continuously scan their microenvironment in order to sense the presence of any endogenous or exogenous danger signals (e.g., damaged cells, microorganisms, etc…). Microglia rapidly respond to these danger signals by orchestrating specific responses that are totally dependent on the nature of such signals, which include the release of cytokines and chemokines, and phagocytosis, in order to clear the factors that trigger these danger cues (Soulet and Rivest, 2008). Microglia react immediately to such signals. However, “activated microglia” is an ambiguous term, as recent findings have demonstrated that these cells are always in an active state while surveying brain microenvironment. This etymology rather outlines microglial cell activity shift from surveying the microenvironment to executing specific immune tasks with diverse functional outcomes. This shift is translated by major changes in microglial cell phenotype under pathological conditions. The actual suggested dichotomy in different microglia phenotypes is mainly based on the M1/M2 activation states of macrophages. Depending on the nature of the stimuli, two functionally different and extreme phenotypes have been proposed for microglia, which are essentially based on *in vitro* assessments. The classically activated M1 phenotype (i.e., pro-inflammatory phenotype), is induced by LPS stimulation, and the alternatively activated M2 phenotype (anti-inflammatory phenotype), is induced by IL4/10 stimulation (Saijo and Glass, 2011). M1 microglia are characterized by the excessive release of several pro-inflammatory cytokines, such as TNFα, IL1β, and IL6, and have a prominent phagocytic capacity (Boche et al., 2013). The alternatively activated M2 microglia are characterized by the important release of several growth and neurotropic factors, such as TGFβ, vascular endothelial growth factor (VEGF), insulin-like growth factor-1 (IGF1), brain-derived neurotrophic factor (BDNF), and nerve growth factor (NGF; Saijo and Glass, 2011; Boche et al., 2013). As such, it has been proposed that a shift toward a M1 phenotype exacerbates the inflammatory response, while a shift toward a M2 phenotype helps in tissue repair and healing (Heneka et al., 2013).

However, recent lessons from macrophages suggested that the same cell has the potential to adopt M1 or M2 phenotype based on either the nature of the stimuli and/or on the original activation status of the cell before being exposed to the stimuli (Mosser and Edwards, 2008). Based on these findings, it has been suggested that it would be more appropriate to consider the phenotype adopted by microglia to be a part of a continuously evolving spectrum of phenotypes, which is dependent on the specific spatiotemporal context under which microglia were investigated (Weitz and Town, 2012). It is noteworthy to mention that most of these studies were performed *in vitro* using simple stimulus, which does not translate the complexity of the microenvironment in which microglia are present *in vivo* (Lampron et al., 2013a). In addition, the implication of some indirect effectors *in vivo*, such as the production of several bioactive molecules by neighboring cells cannot be excluded. As such it is too simplistic to categorize microglial activation into two functionally distinct phenotypes, which depend on the nature of the cerebral microenvironment.

## Author contributions

All authors listed, have made substantial, direct and intellectual contribution to the work, and approved it for publication.

### Conflict of interest statement

The authors declare that the research was conducted in the absence of any commercial or financial relationships that could be construed as a potential conflict of interest.
